# Impact of imposed exercise on energy intake in children at risk for overweight

**DOI:** 10.1186/s12937-016-0206-5

**Published:** 2016-10-21

**Authors:** S. Nicole Fearnbach, Travis D. Masterson, Haley A. Schlechter, Amanda J. Ross, Michael J. Rykaczewski, Eric Loken, Danielle S. Downs, David Thivel, Kathleen L. Keller

**Affiliations:** 1 Department of Nutritional Sciences, The Pennsylvania State University, 110 Chandlee Laboratory, University Park, PA 16802 USA; 2 Department of Kinesiology, The Pennsylvania State University, 276 Recreation Building, University Park, PA 16802 USA; 3 Department of Human Development and Family Studies, The Pennsylvania State University, 119 Health and Human Development Building, University Park, PA 16802 USA; 4 Department of Educational Psychology, University of Connecticut, 328 Charles B. Gentry Building, Storrs, CT 06269 USA; 5 Laboratory of the Metabolic Adaptations to Exercise under Physiological and Pathological Conditions (AME2P), Clermont Auvergne University, EA 3533 Clermont-Ferrand, France; 6 CRNH-Auvergne, 58 Rue Montalembert, 63009 Clermont-Ferrand, France; 7 Department of Food Science, The Pennsylvania State University, 202 Rodney A. Erickson Food Science Building, University Park, PA 16802 USA

**Keywords:** Children, Exercise, Energy intake, Energy expenditure, Energy balance, Obesity

## Abstract

**Background:**

Exercise not only has a direct effect on energy balance through energy expenditure (EE), but also has an indirect effect through its impact on energy intake (EI). This study examined the effects of acute exercise on daily *ad libitum* EI in children at risk for becoming overweight due to family history.

**Methods:**

Twenty healthy-weight children (ages 9–12 years, 12 male/8 female) with at least one overweight biological parent (body mass index ≥ 25 kg/m^2^) participated. Children reported to the laboratory for one baseline and two experimental visits (EX = exercise, SED = sedentary) each separated by 1 week in a randomized crossover design. Two hours into the EX day session, children exercised at 70 % estimated VO_2max_ for 30 min on a cycle ergometer. Objective EI (kcal) was measured at a standard breakfast (~285 kcal) and *ad libitum* lunch, snack and dinner. Meals were identical on the EX and SED days. Activity-related EE (kcal) was estimated with accelerometers worn on the non-dominant wrist and ankle. Relative EI (kcal) was computed as the difference between Total EI and Activity-related EE for each testing day. Paired *t*-tests were performed to test differences in Total EI, Activity-related EE and Relative EI between the EX and SED days.

**Results:**

Across all meals, Total EI was not statistically different between the EX and SED days (*t* = 1.8, *p* = 0.09). Activity-related EE was greater on the EX day compared to the SED day (*t* = 10.1, *p* < 0.001). By design, this difference was predominantly driven by activity during the morning (*t* = 20.4, *p* < 0.001). Because children consumed a similar number of kcal on each day, but had greater Activity-related EE on the EX day, Relative EI was lower (*t* = −5.15, *p* < 0.001) for the EX day (1636 ± 456 kcal) relative to the SED day (1862 ± 426 kcal).

**Conclusions:**

Imposed exercise was effective in reducing Relative EI compared to being sedentary. Morning exercise may help children at risk for becoming overweight to better regulate their energy balance within the course of a day.

## Introduction

Childhood obesity rates have increased substantially over the past forty years, both in the United States and globally [1]. With increasing rates of early onset obesity, children are at the greatest risk for long-term health complications [2] because the disease is often resistant to treatment [3]. The dramatic increase in obesity prevalence has coincided with increased availability of large portions of high energy dense foods, driving a pattern of overeating [4]. In addition, levels of physical activity both in and out of the everyday school setting have decreased, reducing children’s average daily energy expenditure (EE) [5]. Typical methods to reduce EI require intentional energy restriction. These methods may be difficult to sustain as a long-term lifestyle change, particularly during childhood when preferences for sweet, salty and fatty foods are high [6–8]. Therefore, alternative strategies are needed that impact EI and EE to improve obesity outcomes in children. Incorporating exercise as a regular lifestyle component may provide an alternative means to control appetite and energy balance throughout the life course.

The present study aims to understand the acute effects of morning exercise versus rest on daily EI in children ages 9–12 years. Exercise not only has a direct effect on energy balance through EE, but has been shown to also have an indirect effect through its impact on EI [9]. Emerging research has implicated the use of exercise as a preventative measure for overeating and subsequent development of overweight and obesity in adolescents and adults [10–31]. While exercise, relative to rest, has been shown to be effective in reducing subsequent EI and contributing to a negative energy balance in adolescents and adults [17–20, 26–29], limited research has examined this relationship in children under the age of 12 years [32]. Previous work shows that high intensity exercise can induce a state of lower 24-h energy balance by reducing subsequent EI relative to both low intensity exercise and sedentary activity in obese adolescents [27]. This is commonly referred to as the “transient anorexigenic effect” of exercise [33]. In obese adolescents, the greatest effects of high intensity exercise on EI have been seen seven hours post-exercise [27, 34]. These changes were seen without any significant differences in appetite ratings. These findings suggest the use of exercise as a possible strategy to decrease EI, at least in the short term, which could augment attempts to intentionally restrict EI.

Previous studies have focused on the impact of exercise on intake as a treatment strategy in overweight and obese adolescents, but the “anorexigenic effect” of exercise on intake has not been studied in healthy weight children who are at risk for developing obesity due to family history. From a prevention standpoint, it is vital to determine the effectiveness of exercise-related strategies to reduce overeating in children who are predisposed to genetic or environmental factors that promote positive energy balance [35, 36]. This study examined the effects of acute imposed exercise versus imposed sedentariness on daily EI in healthy-weight children with at least one overweight or obese biological parent. Based on previous research with adolescent populations [27], we hypothesized that Total EI and Relative EI (adjusted for Activity-related EE) would be lower on a day with imposed exercise compared to a day where children remained sedentary.

## Methods

### Study design

**Fig. 1:**
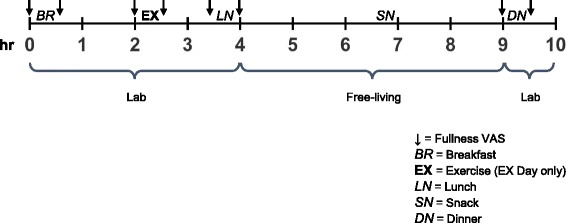
Experimental day (EX or SED) timeline

A within-subjects crossover design study was conducted with a community-based sample of 20 children ages 9–12 years. Children completed 1 baseline and 2 experimental visits (EX = exercise, SED = sedentary in a pre-assigned randomized order) each separated by exactly 1 week. The baseline visit was a four-hour session in the morning to familiarize children with eating in a laboratory environment (breakfast and lunch) and collect baseline measurements (described below). Outside of meal and exercise testing periods, children had access to a series of toys, books, and games and could switch freely between activities. The EX and SED days consisted of the same four-hour morning session in the laboratory, followed by five hours of free-living time at home, and then an in-laboratory dinner session (Fig. 1). Children and their parents received modest financial compensation for their time. This study was approved by the Institutional Review Board of The Pennsylvania State University.

### Participants

Children were recruited using flyers posted in local schools and businesses located around the university. Interested parents completed a phone screening to determine eligibility. Children were considered eligible if by parental report they were normal weight (<85th age- and sex-specific body mass index [BMI] percentile), without food allergies, without medical conditions or contraindications to exercise testing [37], not participating in competitive sports which could skew fitness and exercise test results (year-round or > 3 practice sessions per week), with at least one biological parent who was overweight or obese (BMI > 25 kg/m^2^) [35, 36]. Both child and parent weight status were confirmed by measurement at the baseline visit, described below. One male child was in the overweight category (89th BMI percentile) after baseline measurements, but was retained in the study. This participant did not differ from the group mean in body composition (e.g., % body fat) or on any of the behavioral measures (e.g., food intake, physical activity, exercise test performance). On the first study visit, a parent signed informed consent for their child. Children provided written assent prior to their participation. A total of 20 children were enrolled in the study and completed all three visits. Sample characteristics for these 20 children are listed in Table 1. Sample size calculations (*n* = 20) were derived using G*Power software (version 3.1.9.2) for the primary aim to compare within-subjects differences in EI as a function of condition (EX vs. SED) using paired-sample t-tests [38].

**Table 1:** Participant characteristics (*n* = 20)

	Mean ± SD
Age (years)	10.3 ± 1.1
BMI percentile	41.6 ± 21.7
% body fat	15.6 ± 4.4
	N (%)
Sex	
Male	12 (60)
Female	8 (40)
Race	
White	20 (100)
Non-white	0 (0)

*Abbreviations: BMI* body mass index

### Baseline measurements

#### Anthropometrics & body composition

Prior to breakfast on the baseline visit, anthropometrics (height and weight) were measured to the nearest 0.1 cm and 0.1 kg by a trained researcher. Children and their parents were each weighed and measured twice using a standard scale (Detecto model 437, Webb City, MO) and stadiometer (Seca model 202, Chino, CA) in light clothing and stocking feet. Height (m) and weight (kg) were converted to body mass index (BMI; kg/m^2^) for the parent, and age- and sex-specific BMI z-score and BMI percentile for the child using the Centers for Disease Control and Prevention conversion program [39]. Percent body fat was measured using bioelectrical impedance analysis (Tanita model BF-350, Arlington Heights, IL, USA) [40].

#### Fitness testing

Two hours into the baseline visit, children completed the YMCA graded submaximal cycle test to estimate cardiorespiratory fitness [41]. Children were outfitted with a Polar Heart Rate Transmitter chest strap and wrist unit receiver (Polar Electro Inc. model T31-Coded, Lake Success, NY, USA). Participants remained seated for five minutes while a researcher explained the procedure and instructed the child on the use of the Borg Scale for Ratings of Perceived Exertion (RPE) [42]. At the end of the five minute period, a supervising nurse obtained resting heart rate and blood pressure measurements. Children were then familiarized with the cycle ergometer (Lode Corival V2, Lode Holding BV, Groningen, The Netherlands) and completed a three-minute warm-up, followed by the YMCA submaximal cycle test. The YMCA cycle test follows a branching, multi-stage format (Table 2) to determine the relationship between heart rate and work rate in order to estimate the individual’s VO_2max_. Children are required to pedal at a constant rate (50 ± 2 rpm) while researchers adjust the resistance (i.e., work rate) on the cycle ergometer at each stage. Heart rate values are recorded every minute, while blood pressure and RPE are measured every three minutes. The test requires that each participant completes two separate workload stages that result in steady-state heart rates between 110 and 150 beats per minute. Steady-state is achieved when two consecutive heart rate values are within ±5 beats per minute. VO_2max_ was estimated using the graph plot and extrapolation technique [41, 43]. This estimated VO_2max_ was used to determine the work rate for the 70 % intensity cycle test on the EX Day (described below).

**Table 2:** YMCA Submaximal Cycle Ergometer Test protocol; American College of Sports Medicine [36]

Stage 1	150 kg^.^m^.^min^−1^ 0.5 kg 24 W			
	HR: < 80	HR: 80–89	HR: 90–100	HR: > 100
Stage 2	750 kg^.^m^.^min^−1^ 2.5 kg 123 W	600 kg^.^m^.^min^−1^ 2.0 kg 98 W	450 kg^.^m^.^min^−1^ 1.5 kg 73 W	300 kg^.^m^.^min^−1^ 1.0 kg 49 W
Stage 3	900 kg^.^m^.^min^−1^ 3.0 kg 147 W	750 kg^.^m^.^min^−1^ 2.5 kg 123 W	600 kg^.^m^.^min^−1^ 2.0 kg 98 W	450 kg^.^m^.^min^−1^ 1.5 kg 73 W
Stage 4	1050 kg^.^m^.^min^−1^ 3.5 kg 172 W	900 kg^.^m^.^min^−1^ 3.0 kg 147 W	700 kg^.^m^.^min^−1^ 2.5 kg 114 W	600 kg^.^m^.^min^−1^ 2.0 kg 98 W

*Abbreviations: HR* heart rate (beats per minute), *kg* kilograms, *m* meter, *min* ^*−1*^ per minute, *W* Watts

### 70 % intensity exercise protocol

Two hours into the EX day session (Fig. 1), children completed the cycle ergometer exercise test. Participants were outfitted with the heart rate monitor, and resting heart rate and blood pressure measurements were taken. After a three-minute warm-up, children exercised at their individual 70 % estimated VO_2max_ for 30 min. The starting work rate (in Watts) was determined from the linear association between heart rate and work rate established during the submaximal exercise test [41]. The work rate was either confirmed or adjusted throughout the test to maintain a target heart rate between 70-80 % age-predicted maximum heart rate (e.g., 10-year-old: 147–168 beats per minute). Children could request water at any point during the test. Researchers and the attending nurse encouraged children throughout the exercise protocol with positive verbal cues, cheering and clapping. After completion of the exercise test, participants had a five-minute cool-down period on the bike, followed by ten minutes of light stretching.

### Accelerometer measurements

Children wore an ActiGraph GT3X-BT accelerometer on their non-dominant wrist for 10 h on each testing day (EX, SED). In addition, children wore a second accelerometer on their non-dominant ankle for the YMCA submaximal cycle test and the 30-min exercise test (70 % individual estimated aerobic capacity) to more accurately measure activity in the seated position on the cycle ergometer. We used this hybrid measure to estimate Activity-related EE [44]. The 4 h during the morning session were considered in-laboratory time, while the 6 h in the afternoon were considered free-living time. Activity-related EE was extracted for each child for the entire 10-h period, and then separately for the morning (in-lab) versus the afternoon (free-living). All data were validated and scored in ActiLife 6 software (ActiGraph, LLC, Pensacola, FL, USA) using Freedson Combination (1998) to calculate Activity-related EE.

### Food intake measurement

#### Test-meal procedures

Children arrived to the laboratory after an overnight fast on all three testing days. Objective EI (kcal) was measured at a standard breakfast and lunch, snack and dinner test-meals. Meals were identical on the EX and SED days. Fullness ratings were completed before and after each laboratory meal on a vertical 150 mm visual analog scale (VAS) referred to as “Freddy Fullness” (data not shown) [45]. On the first visit, children conducted taste tests to report liking and wanting for each breakfast, lunch and snack food on VAS. On the second visit, children tasted and rated liking and wanting on VAS for each dinner food (data not shown). Timing for the meals and VAS ratings is depicted in Fig. 1.

##### Breakfast

All children were required to consume a standardized breakfast on all three test days consisting of an English muffin toasted with one tablespoon butter, banana and orange juice (285 kcal total) (Table 3). Children’s liking for and willingness to eat the breakfast foods were confirmed at screening, prior to the first visit. Children were considered to have finished the meal if they consumed ≥ 95 % of each individual food item within 30 min (average duration = 11 ± 5 min). All 20 children met these requirements at each of the three breakfast meals.

**Table 3:** Food items, serving sizes and calorie contents for each meal. Abbreviations: g, grams; fl oz., fluid ounces; kcal, kilocalories

Breakfast Food Item	Serving Size	Kcal per Serving
English muffin (with butter)	1 muffin + 1 tablespoon butter	151
Banana (without peel)	60 g	51
Orange juice	178 g (6 fl oz.)	83
Total		285
Lunch Food Item	Serving Size	Kcal per Serving
Wheat bread	2 slices	170
Deli meat (pick one)		
Ham	114 g	100
Turkey	95 g	100
+ Cheese (pick one)		
Cheddar	42 g	157
Provolone	38 g	140
American	53 g	151
OR		
Peanut butter	30 g	190
+ Jelly	36 g	60
Pretzels	42 g	165
OR		
Baked chips	39 g	65
Apple slices	102 g	53
OR		
Grapes	77 g	53
Carrots	100 g	41
OR		
Tomatoes	140 g	41
Ranch dip	30 g	140
Brownies (3)	43 g	188
Total		997–1014
Snack Food Item	Serving Size	Kcal per Serving
Chocolate chip granola bar	1 bar	104
Mixed fruit cocktail	117 g	62
Apple juice	250 g (8 fl oz.)	136
Total		302
Dinner Food Item	Serving Size (multiple servings available)	Kcal per Serving
Macaroni & cheese	400 g	551
Garlic bread	75 g	270
Broccoli (with butter)	120 g	53
Applesauce	128 g	110
Cookies (3)	46 g	243
Total		1,227

##### Lunch

Prior to the first visit, children were given the opportunity to select from a pre-set menu of available items for lunch. Children chose a sandwich (peanut butter & jelly or deli meat & cheese), vegetable (carrots or tomatoes) with ranch dip, fruit (apple slices or grapes), and salty snack (pretzels or baked chips). All children also received brownies and a bottle of water with their lunch. All serving sizes were controlled to ensure that any combination of food items provided approximately the same number of total calories (997–1014 kcal), which provided > 50 % of children’s caloric needs for the day. Possible food choices and serving sizes are reported in Table 3. Children were instructed that they had up to 30 min to eat freely from the food items provided. If they were finished before the 30 min had elapsed, they notified a researcher (average duration = 19 ± 6 min).

##### Snack

The snack consisted of a granola bar, fruit cocktail and juice (~302 kcal) (Table 3). All foods were pre-weighed and packed for children to take home during free-living time on the EX and SED days. Parents were given written and verbal instructions to provide the snack at a set time and to return any packaging and uneaten food items to researchers upon arrival for dinner. Additional written and verbal instructions were given to not eat or drink anything except water before the snack, or for the 2 h prior to dinner. Compliance was checked by sending text message reminders to parents at snack time and requesting a response. These messages asked parents to remind children that they could eat as much as they wanted of any of the snack foods. Upon return, packaging was re-weighed to measure snack intake.

##### Dinner

On the EX and SED days, children reported to the laboratory at least 2 h fasted for an *ad libitum* dinner test-meal consisting of macaroni and cheese, garlic bread, broccoli, applesauce and cookies (~1227+ kcal) (Table 3). Children were instructed that they had up to 30 min to eat as much as they wanted from the available foods. They were also able to request additional servings of the foods at this meal. A researcher was available in the room during the meal and prompted the child if they finished a serving of a particular food. Children could also notify the researcher if they finished eating before the 30 min had elapsed (average duration = 19 ± 5 min).

#### Nutrient analysis

Pre- and post-meal weights for each food item were measured to the nearest 0.1 g, and used to calculate intake in grams. This was later converted to EI (kcal) by meal (Breakfast, Lunch, Snack and Dinner) and in SPSS Statistics (Version 22; IBM Corporation, Armonk, NY, USA) using nutrition label information. Total EI (kcal) was computed as the sum of EI from each of the individual meals (Breakfast, Lunch, Snack and Dinner EI). Macronutrient intake (kcal) was also calculated for each day using nutrition label information for each food item, summed across each testing day. Relative EI (kcal) was computed as the difference between Total EI and Activity-related EE for each testing day.

### Statistical analysis

Descriptive statistics for participant characteristics (i.e., means and standard deviations on continuous variables and frequencies on categorical variables) were calculated on the full sample. Pearson’s correlations were computed to determine the association between Total EI on the EX and SED days. Paired *t*-tests were performed to test differences in Total EI, EI by meal (standard breakfast, lunch, snack and dinner EI), macronutrient intake, Activity-related EE (total, morning, afternoon) and Relative EI between the EX and SED days. Effect sizes (Cohen’s *d*) were calculated for all paired *t*-test results. Data were analyzed using SPSS. Test results were considered significant at *p* < 0.05.

## Results

Across all meals, Total EI was not statistically different between the EX and SED days (*t* = 1.8, *p* = 0.09). Total intake on the two days was highly correlated (*r* = 0.93, *p* < 0.01). By design, EI at the standard breakfast was not different between the two days (*t* = 0.2, *p* = 0.87). In addition, EI at the lunch (*t* = 2.0, *p* = 0.06), snack (*t* = −1.9, *p* = 0.08), or dinner (*t* = 1.2, *p* = 0.24) did not differ on the EX day versus the SED day. In regards to macronutrient intake, energy intake from protein (*t* = 2.1, *p* < 0.05) and fat (*t* = 2.3, *p* < 0.05) were higher on the EX day compared to the SED day, with no differences in carbohydrate intake (*t* = 1.2, *p* = 0.24). However, this did not result in a significant difference in the proportion of calories from each macronutrient (all *p* > 0.05, data not shown) between the two testing days.

Activity-related EE was greater on the EX day compared to the SED day (*t* = 10.1, *p* < 0.001). This difference was predominantly driven by in-laboratory activity during the morning (*t* = 20.4, *p* < 0.001). Afternoon free-living Activity-related EE was not different between the two days (*t* = 1.8, *p* = 0.09).

**Fig. 2:**
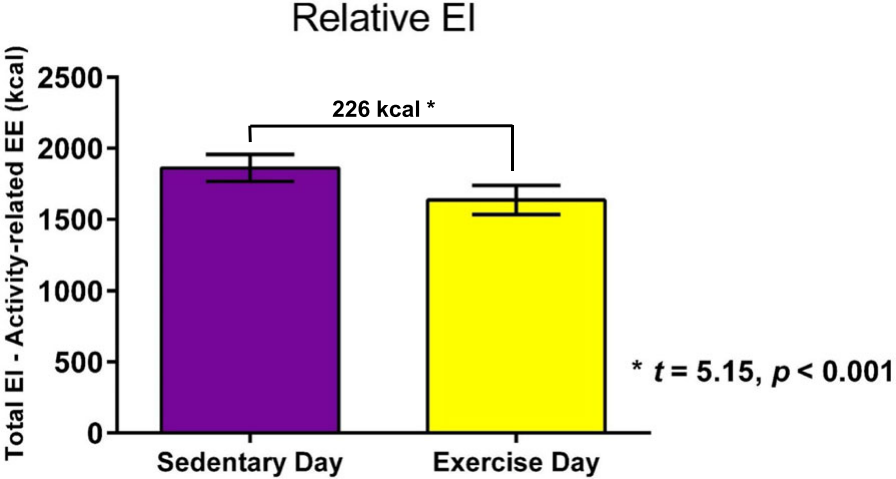
Relative EI (kcal) was 226 kcal lower on the EX Day (1636 ± 456 kcal) compared to the SED Day (1862 ± 426 kcal); *t* = 5.15, *p* < 0.001

Because children ate a similar number of kcal on each day, but had greater Activity-related EE on the EX day, Relative EI was lower (*t* = −5.15, *p* < 0.001) for the EX day (1636 ± 456 kcal) compared to the SED day (1862 ± 426 kcal) (Fig. 2). In other words, Total EI adjusted for Activity-related EE was 226 kcal lower on the EX Day than the SED day. Paired *t*-test results for EI and EE variables and effect sizes for these results are summarized in Table 4.

**Table 4:** Results of paired *t*-tests for mean comparisons of energy intake (EI) and energy expenditure (EE) variables between the Exercise (EX) and Sedentary (SED) Days

	EX Day	SED Day	Paired Diff. (± SD)	*t*	*p*	*Effect size (d)*
Total EI (kcal)	2171 ± 566	2088 ± 497	83 ± 204	1.8	0.09	0.41
Breakfast EI (kcal)	287 ± 8.2	286 ± 8.5	0 ± 9	0.2	0.87	0.04
Lunch EI (kcal)	683 ± 186	639 ± 193	45 ± 100	2.0	0.06	0.45
Snack EI (kcal)	245 ± 72	256 ± 61	−12 ± 28	−1.9	0.08	0.43
Dinner EI (kcal)	956 ± 362	907 ± 310	50 ± 182	1.2	0.24	0.27
EI from Macronutrients (kcal)						
Carbohydrate (kcal)	1269 ± 337	1235 ± 295	33 ± 122	1.2	0.24	0.85
Protein (kcal)	207 ± 68	196 ± 67	11 ± 23	2.1	0.04 *	1.48
Fat (kcal)	694 ± 181	655 ± 155	39 ± 76	2.3	0.03 *	1.61
Total Activity-related EE (kcal)	534 ± 263	226 ± 146	308 ± 137	10.1	0.001*	2.25
Morning Activity-related EE (kcal)	394 ± 123	116 ± 77	278 ± 61	20.4	0.001*	4.56
Afternoon Activity-related EE (kcal)	144 ± 147	110 ± 74	33 ± 86	1.8	0.09	0.40
Relative EI (kcal)	1636 ± 456	1862 ± 426	−226 ± 196	−5.15	0.001 *	1.15

*Significance at *p* < 0.05

## Discussion

The purpose of this pilot study was to examine the effects of acute exercise on daily EI in healthy-weight 9–12 year-old children who were at risk for becoming overweight due to family history. Based on previous research on adolescents with obesity [27], we hypothesized that Total EI and Relative EI (Total EI adjusted for Activity-related EE) would be lower on the EX day compared to the SED day. In line with our hypothesis, we did find that Relative EI was 226 kcal lower on the EX day compared to the SED day, indicating a beneficial effect of imposed exercise on children’s energy balance over the course of a day. This finding was a result of the fact that children did not significantly adjust their daily EI to fully compensate for differences in activity-related EE between the two days. Participants consumed essentially the same number of calories (within 85 kcal) on the EX day and the SED day, but had greater Activity-related EE (~310 kcal) on the EX day than the SED day. There were small but statistically significant increases in calories consumed from protein (11 kcal) and fat (39 kcal) on the EX day relative to the SED day. In sum, we found that imposed exercise had a beneficial impact on daily energy balance in children who are at risk for becoming overweight by increasing Activity-related EE without a significant increase in subsequent EI.

In the current study, we looked to extend previous findings in adolescents with obesity regarding the “anorexigenic effect” of exercise on subsequent food intake [27]. We used a similar design to previous studies to examine the effects of exercise in a younger age group of pre-adolescent children who were at risk for becoming overweight. We found that imposed exercise was effective in reducing Relative EI when compared to the SED day. We did not find evidence of a compensatory response to the exercise bout in regards to children’s EI at subsequent meals. Children consumed approximately the same number of calories on both days, demonstrating that they did not adjust their EI to match differences in Activity-related EE between the EX day and the SED day. In other words, the short-term benefits of exercise on EE were not immediately offset by compensatory EI in our sample of healthy weight children. Previous studies in adolescents and adults have shown mixed results for acute post-exercise EI [33, 46], which can be attributed to differences in participant characteristics (e.g., lean versus obese) or exercise methodologies (e.g., intensity, modality). Generally, subsequent EI is not greater after exercise relative to rest in healthy populations, whether at a single meal or over the course of a day [30, 46]. Once differences in EE from exercise are accounted for, Relative EI is typically lower after exercise versus a control condition [23, 27, 31]. These results, together with ours, suggest that morning moderate-to-vigorous intensity exercise may be an effective strategy to prevent positive energy balance in children at risk for becoming overweight, at least in the short term. Additional longer-term studies are needed to assess the effectiveness of structured morning exercise for childhood obesity prevention.

It is important to note that the timing of the exercise bout relative to the meals can affect subsequent EI, but previous studies disagree regarding the most effective time interval to reduce EI. A recent study in healthy weight adolescent males demonstrated that exercise immediately prior to lunch was more effective in reducing lunch EI when compared to an identical exercise bout approximately 2 h prior to lunch [11]. There are several proposed mechanisms for the effects of acute exercise on appetite and EI regulation. Exercise can act indirectly on energy balance through influences on body composition (e.g., fat and lean mass), gut peptide signaling (e.g., polypeptide YY 3–36, glucagon-like peptide-1) and brain responses to food-related cues [9, 47]. These signals, in theory, impact appetite and subsequent food intake [9, 47]. Given the short half-life of many appetite-regulating hormones, it may be expected that a shorter delay between an exercise bout and a meal is more effective in reducing EI than a longer interval. In the current study, the delay between the exercise bout and the lunch test-meal was approximately 45 min. We did not find a significant difference in lunch EI between the EX and SED days. Other studies have found lasting effects of acute morning exercise on EI later in the day. For example, Thivel and colleagues found that high intensity exercise was effective in reducing intake at a dinner test-meal 7 h post-exercise [27]. The mechanism for these more sustained effects is unclear, but important to investigate given that obesity prevention requires chronic regulation of energy balance.

One major methodological difference in our study, versus previous studies [27], is that EE was not matched across the two days (EX versus SED). Activity-related EE was controlled during the four hours of in-laboratory time in the morning, but we also allowed children free-living time in the afternoon. This allowed us to test whether children would compensate for the morning exercise by increasing sedentary time later in the day. We did not find any differences in afternoon Activity-related EE between the EX and SED days. In other words, children were not less active following the exercise bout than they were following imposed sedentary time. The lack of a compensatory response in our sample is suggestive of one promising behavioral attribute that could help children maintain a healthy weight. A recent study in overweight boys found that after a vigorous exercise session, the participants spontaneously decreased their physical activity EE during the following 24 h [48]. Participation in structured exercise may be a more effective strategy in healthy weight populations with fewer negative consequences on subsequent leisure-time physical activity. A systematic review in healthy adults found minimal evidence that prescribed exercise affects non-exercise physical activity and EE [49]. Further research in children is warranted to determine which characteristics make an individual more or less likely to compensate for imposed exercise.

This preliminary study represents a novel application of the working model of energy balance in a sample of children at risk for becoming overweight. There are several strengths of this study. First, we have objective measures of daily EI across multiple test-meals during which we were able to assess children’s energy intake. Objective intake measures are advantageous compared to self-reported EI, a method which introduces misreporting biases [50]. In addition, identical meals across the two experimental days allowed us to look specifically at the within-subjects effect of the exercise bout on Total EI. Another strength is the inclusion of objective measures of Activity-related EE, both in the laboratory and free-living. Our study was designed to facilitate acclimation to the laboratory environment (research personnel, exercise testing and test-meals) at the baseline visit. This helped to reduce the novelty effect on our outcome variables on the experimental days. Finally, we had a 100 % retention rate and full reported and/or observed compliance with instructions and protocols across the three testing days.

Some limitations of the current study should be noted. We were not able to assess intake outside of the 10-h experimental day and are unsure whether children’s EI differed in the late evening (e.g., dessert) or the following day. While there were no reports of additional food or beverage intake during the free-living period between lunch and dinner, we acknowledge that our compliance rates with food-related study procedures are subject to the biases of self-report. In addition, a few of the differences in intake between the two days were non-significant trends (*p* < 0.10), including lunch EI (*p* = 0.06). Post-hoc analyses demonstrated we were underpowered (Power < 0.30) to detect differences in intake of individual meals/snack between the two days. It is possible that these differences would be significant with a larger sample size. Our sample was homogenous in demographic characteristics, which limits the generalizability of our findings. In addition, our classification of “at risk for becoming overweight” was solely based on parent weight status [35, 36], and not inclusive of additional genetic, family or environmental-level factors that can impact energy balance-related behaviors [51]. Future studies may be able to identify more specific phenotypes of obesity risk in the recruitment phase. Finally, this study did not include measures of resting metabolic rate or thermic effect of food. Therefore, we cannot evaluate effects of exercise on total daily EE or overall energy balance.

## Conclusions

Children in this study did not adjust their EI to match differences in Activity-related EE, resulting in relative positive energy balance on the SED compared to the EX day. Imposed exercise may help children at risk for becoming overweight better regulate their food intake within the course of a day. In order to allow for more personalized prevention strategies, future research is necessary to determine the individual-level child characteristics that are likely to impact the effect of exercise on energy intake in this population.

## Abbreviations

*ad lib*: *ad libitum*
BMI: Body mass index
EE: Energy expenditure
EI: Energy intake
EX: Exercise
kcal: Kilocalories
RPE: Ratings of perceived exertion
SED: Sedentary
VAS: Visual analog scale
VO_2max_: Maximal aerobic capacity
